# Supplementary material for: MASLD-related HCC: Multicenter study comparing patients with and without cirrhosis
Source: JHEP Rep. 2024 Jun 29;6(10):101160. doi: 10.1016/j.jhepr.2024.101160 (PMC11474187; doi:10.1016/j.jhepr.2024.101160)

MASLD-related HCC: Multicenter study comparing patients with and without cirrhosis

Carole Vitellius, Elvire Desjonqueres, Marie Lequoy, Giuliana Amaddeo, Isabelle Fouchard, Gisele N'Kontchou, Clemence M Canivet, Marianne Ziol, Hélène Regnault, Adrien Lannes, Frederic Oberti, Jerome Boursier, Nathalie Ganne-Carrie

Table of contents

Table S1 2

Fig. S1 3

Fig. S2 4

Fig. S3 5

Fig. S4 6

Fig. S5. 7

Table S1: Predictors of overall survival in univariate analysis

	All (n=354)	Alive (n=89)	Dead (n=265)	p
Centre (%):				0.435
Angers	48.6	42.7	50.6	
Bondy	25.7	27.0	25.3	
Creteil	13.0	13.5	12.8	
Paris Saint Antoine	12.7	16.9	11.3	
Age (years)	73.0 (66.0–79.0)	71.0 (62.0–77.0)	73.0 (68.0–80.0)	<0.001
Male sex (%)	78.0	77.5	78.1	0.715
BMI (kg/m^2)	29.4 (26.1–33.0)	28.9 (26.1–32.7)	29.6 (26.1–33.1)	0.441
Diabetes (%)	71.5	69.6	72.2	0.362
Arterial hypertension (%)	80.8	80.0	81.1	0.980
Underlying cirrhosis (%)	65.0	53.9	68.7	0.043
Platelets (G/l)	173 (116–247)	186 (118–248)	172 (116–247)	0.177
Bilirubin (µmol/l)	13 (9–20)	10 (7–15)	13 (9–22)	<0.001
Prothrombin time (%)	83 (72–94)	85 (75–96)	82 (72–93)	0.016
Creatinine (µmol/l)	77 (65–94)	76 (65–88)	78 (65–96)	0.035
AFP (ng/ml)	9 (4–176)	5 (3–14)	16 (5–381)	0.002
AFP (%):				<0.001
≤100 ng/ml	69.8	86.7	64.0	
101–1000 ng/ml	14.8	9.6	16.5	
>1000 ng/ml	15.4	3.6	19.4	
Number of lesions (%):				<0.001
1	52.9	67.8	47.8	
2–3	24.9	25.3	24.7	
≥4	22.2	6.9	27.5	
Size of the largest lesion (%):				<0.001
<3 cm	34.0	47.7	28.3	
3–5 cm	28.5	30.2	27.8	
≥5 cm	37.5	22.1	43.9	
Portal vein tumor thrombosis (%)	20.7	6.7	25.5	<0.001
Extra-hepatic metastasis (%)	10.3	3.4	12.6	<0.001
BCLC classification (%):				<0.001
0	8.9	15.9	6.5	
A	41.1	63.6	33.5	
B	19.3	12.5	21.5	
C	18.7	6.8	22.7	
D	12.1	1.1	15.8	

BMI: body mass index, AFP: alpha-foetoprotein

Statistical comparisons between the groups were performed using the univariate Cox Model for quantitative variables and log-rank test for qualitative variables.

Fig. S1: Overall survival in the study population

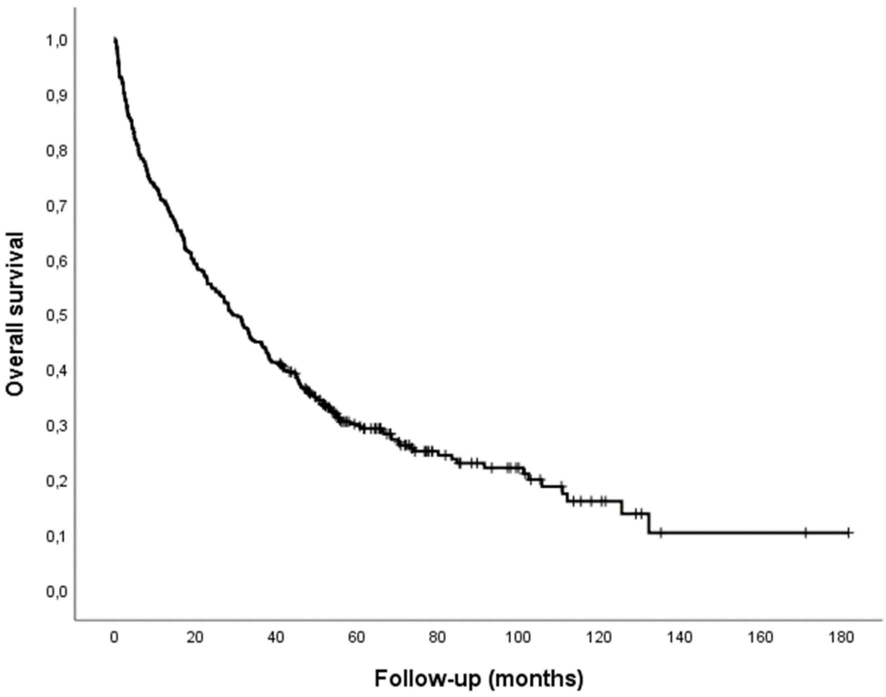

Fig. S2: Overall survival according to treatment modalities for hepatocellular carcinoma

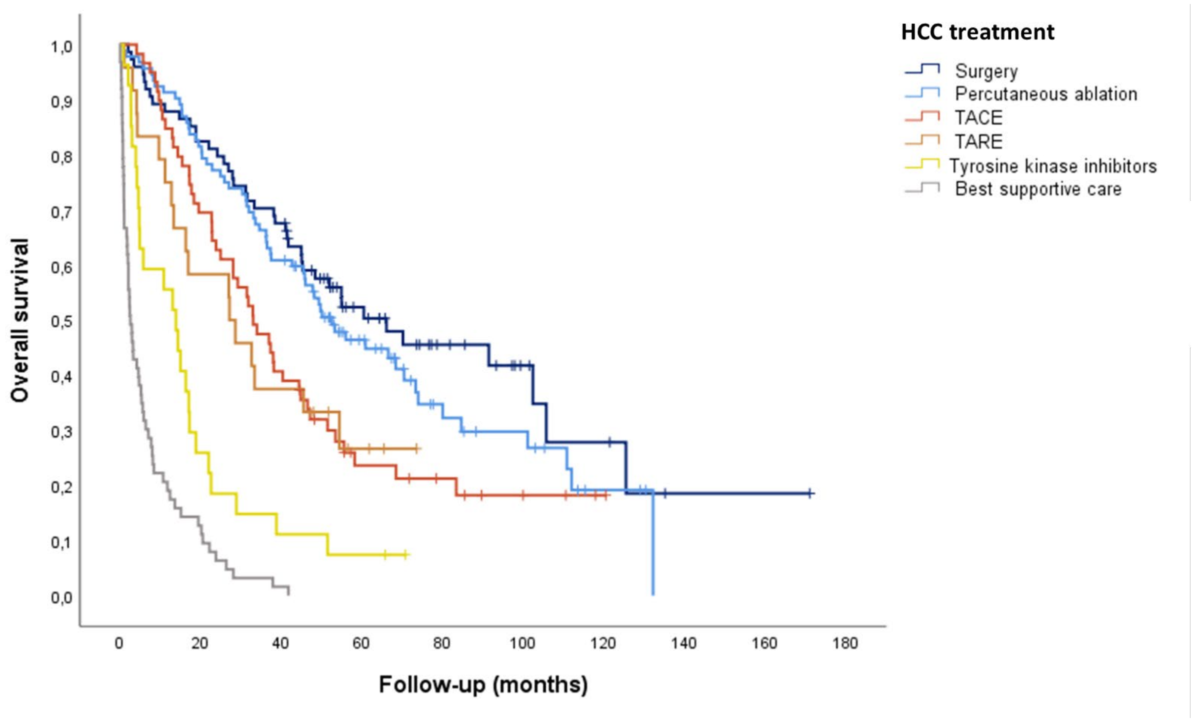

Fig. S3: Treatment modalities for hepatocellular carcinoma according to BCLC classification stages

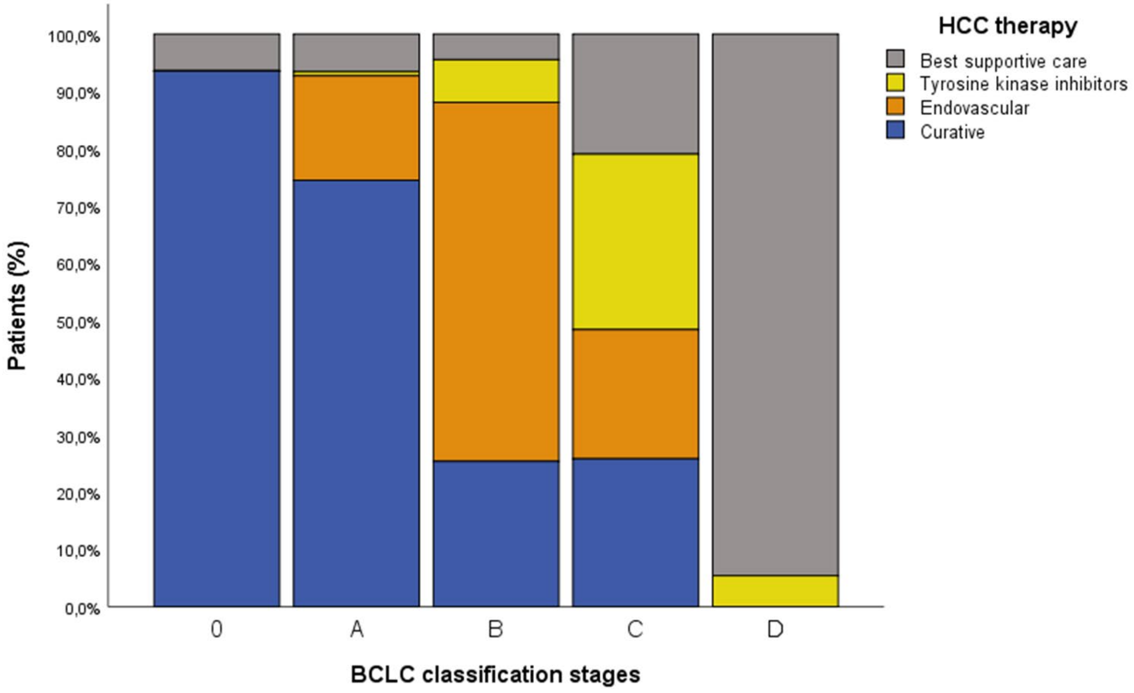

Fig. S4: Overall survival in patients undergoing surgery according to cirrhosis status

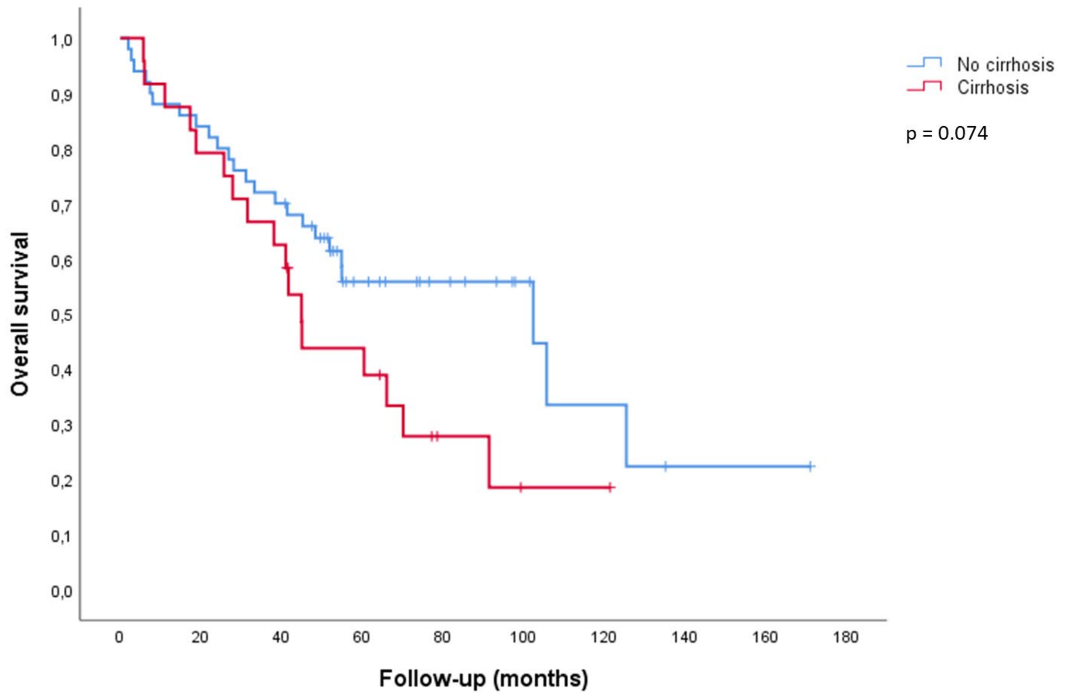

Statistical comparison between the cirrhotic and non-cirrhotic patients was conducted using the log-rank test.

Fig. S5: Number of hepatocellular carcinomas newly presented at the multidisciplinary liver tumor meeting of the Angers University Hospital between 2007 and 2018 (panel s5a), and trends in the causes of chronic liver disease associated with hepatocellular carcinoma in the Angers center (panel s5b).

a

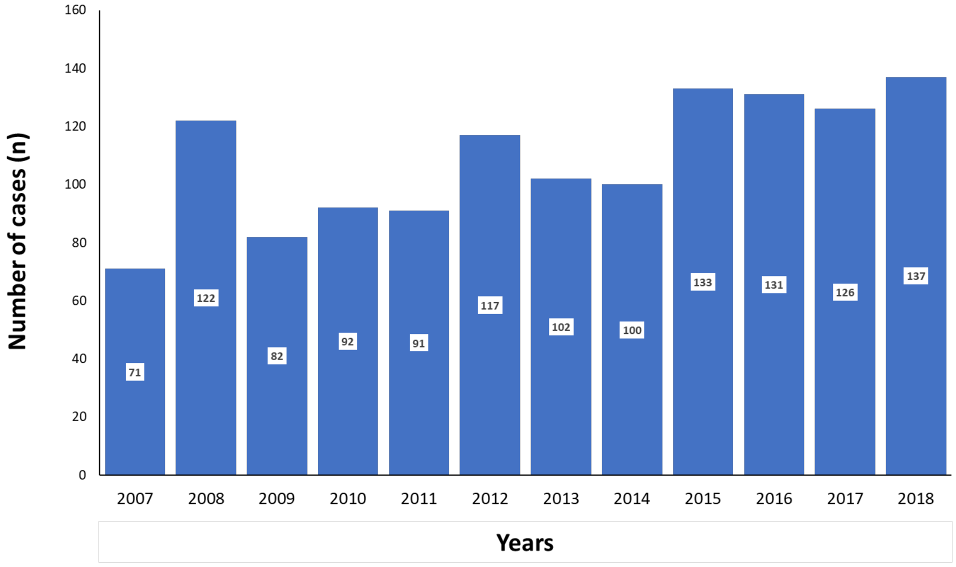

b

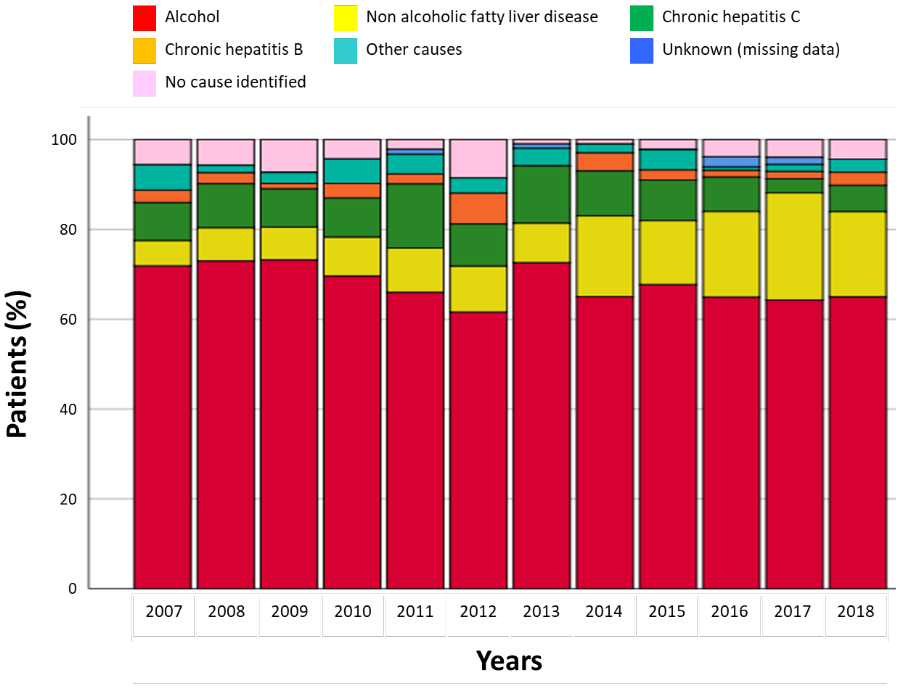

Supplement: Multimedia component 1 [file mmc1.pdf]
