# Supplementary material for: MASLD-related HCC: Multicenter study comparing patients with and without cirrhosis
Source: JHEP Rep. 2024 Jun 29;6(10):101160. doi: 10.1016/j.jhepr.2024.101160 (PMC11474187; doi:10.1016/j.jhepr.2024.101160)

# MASLD-related HCC: Multicenter study comparing patients with and without cirrhosis

**Carole Vitellius**[1,2,*,†], **Elvire Desjonqueres**[3,†], Marie Lequoy[4], Giuliana Amaddeo[5], Isabelle Fouchard[1,2], Gisele N'Kontchou[3], Clemence M. Canivet[1,2], Marianne Ziol[6,7], Hélène Regnault[5], Adrien Lannes[1,2], Frederic Oberti[1,2], Jerome Boursier[1,2,‡], Nathalie Ganne-Carrie[3,7,‡]

JHEP Reports **2024**. vol. 6 | 1–8

Check for updates

**Background & Aims:** Despite its growing incidence, hepatocellular carcinoma (HCC) related to metabolic dysfunction-associated steatotic liver disease (MASLD) in non-cirrhotic livers remains poorly characterized. We compared the characteristics, management, survival, and trends of MASLD-related HCC in patients with or without underlying cirrhosis in a large multicenter cohort.

**Methods:** A total of 354 cases of MASLD-related HCC presented at the liver tumor meetings of four French university hospitals between 2007 and 2018 were included in the study. Data were extracted from the meetings' databases and from the French Birth and Death Registry.

**Results:** Of HCC cases, 35% occurred in the absence of cirrhosis. HCC was diagnosed through screening in 60% of patients with cirrhosis, and incidentally in 72% of patients without it. Patients without cirrhosis were older, had a greater tumor burden, but also better liver function than patients with cirrhosis. Patients without cirrhosis showed better overall survival than those with cirrhosis ($p$ = 0.043). However, cirrhosis was not independently associated with overall survival, the independent predictors were age, liver function, tumor burden and BCLC classification. Patients without cirrhosis underwent surgery more frequently than patients with cirrhosis (41% *vs.* 11%, $p$ <0.001), even in cases where the largest tumors were ≥5 cm (42% *vs.* 14%, $p$ = 0.002) or there were four or more lesions (19% *vs.* 2%, $p$ = 0.024). Among the patients (with/without cirrhosis) who underwent surgery, survival was not significantly different. The cirrhosis/no cirrhosis ratio remained stable over the study period.

**Conclusions:** In MASLD-related HCC, patients without cirrhosis account for 35% of cases and have poor prognostic factors (higher age and larger tumors) but also better liver function, resulting in more aggressive management of advanced tumors and better survival compared to patients with cirrhosis.

© 2024 The Author(s). Published by Elsevier B.V. on behalf of European Association for the Study of the Liver (EASL). This is an open access article under the CC BY license (http://creativecommons.org/licenses/by/4.0/).

## Introduction

Liver cancer is the sixth most-frequent cancer and the third leading cause of cancer-related death worldwide.[1] Hepatocellular carcinoma (HCC) represents 85–90% of all primary liver cancers. The main causes of underlying chronic liver disease in HCC are alcohol, viral hepatitis, and non-alcoholic fatty liver disease (NAFLD),[2] the nomenclature of the latter having very recently evolved to metabolic dysfunction-associated steatotic liver disease (MASLD).[3] The growing epidemics of obesity and type 2 diabetes have nearly doubled the prevalence of MASLD, from 20% in 2000–2005 to 38% in 2016–2019.[4,5] Close to 25% of patients with MASLD develop metabolic dysfunction-associated steatohepatitis, which can lead in turn to cirrhosis and HCC.[6] Consequently, the incidence of HCC related to MASLD is projected to increase by 47% to 130% by year 2030.[7]

The literature provides conflicting information on MASLD-related HCC. An analysis of a large registry from the United States showed higher age, higher tumor burden, and a lower rate of curative treatment for MASLD-related HCC compared to non-MASLD-related HCC.[8] A recent meta-analysis also reported higher age and larger tumor diameter in MASLD-related HCC, but, in contrast to the US registry, it provided a high, 65% rate of curative treatment.[9] In another work, despite less HCC-specific treatment, 1-year survival in MASLD-related HCC did not differ from that in non-MASLD-related HCC.[10] MASLD-related HCC is peculiar in that it occurs in the absence of cirrhosis in around 40% of patients.[9,11] HCC without underlying cirrhosis lies outside the scope of screening, which could explain late diagnosis, more advanced cancer, and less possibility for curative therapy. However, the absence of cirrhosis may also permit more aggressive treatment because of preserved liver function and absence of portal hypertension. In the present work and to explore these aspects, we sought to compare the characteristics, management, survival, and trends

* Corresponding author. Address: Dr Carole Vitellius; Service d'Hépato-Gastroentérologie, CHU 49933 Angers Cedex 09, France; Tel.: (33) 2 41 35 34 10, fax: (33) 2 41 35 41 19.
*E-mail address:* Carole.Vitellius@chu-angers.fr (C. Vitellius).
† Equally contributed to this work as first authors
‡ Equally contributed to this work as senior authors
https://doi.org/10.1016/j.jhepr.2024.101160

of MASLD-related HCC in patients with or without underlying cirrhosis in a large multicenter cohort.

## Patients and methods

### Patients

Consecutive cases of MASLD-related HCC presented at the multidisciplinary liver tumor meetings of four French university hospitals (Angers, Bobigny, Creteil, Paris Saint Antoine) were retrospectively included in the present work. The inclusion period extended from January 2007 to December 2018 for the Angers and Bobigny centers, and from January 2012 to December 2018 for the Creteil and Paris Saint Antoine centers. The cause of the underlying liver disease (alcohol, chronic viral hepatitis, NAFLD, haemochromatosis, *etc*.) was available in the electronic file of the multidisciplinary liver tumor meetings, but not all the components of the NAFLD or MASLD definitions were available. It was therefore not possible to precisely determine if patients belonged to one, the other, or both definitions. Nevertheless, all patient presentations and electronic file completions were made by specialized hepato-gastroenterologists, including the patients' referring physician, which ensured an accurate qualification of the underlying liver disease as of metabolic origin. This diagnosis was based on the presence of metabolic risk factors with no other known concomitant cause of chronic liver disease (excessive alcohol consumption >20 g/day in women or >30 g/day in men, chronic viral hepatitis, hemochromatosis, auto-immune liver disease). Several studies have shown there is almost complete overlap between NAFLD and MASLD.[12] Therefore, to be in accordance with the new nomenclature, we qualified the patients included in the study as having MASLD.

In instances where a case was presented more than once at the meeting, only the first presentation was considered. The study was approved by the Local Ethics Committee of Avicenne Hospital (Bobigny; CLEA-2021-213).

### Data collection

Data were collected from the databases of the multidisciplinary liver tumor meetings. Any missing data were retrieved from the patients' medical records.

#### *Baseline data*

Data collected at baseline were as follows: clinical characteristics (age, gender, body mass index, diabetes, arterial hypertension), circumstances of HCC diagnosis (incidental, pain, complication, or screening), blood parameters (platelets, bilirubin, prothrombin time, creatinine, alpha-foetoprotein), radiology (uni or bilobar localization, number of HCC lesions, size of the largest HCC lesion, portal vein tumor thrombosis, extrahepatic metastasis), and histology (fibrosis stage according to the NASH CRN classification[13]). The diagnosis of HCC was established either by histological examination performed by an experienced pathologist or by the use of international non-invasive criteria (AASLD and/or EASL) in force at the time of diagnosis and validated at the multidisciplinary liver tumor meetings. The Barcelona Clinic Liver Cancer (BCLC) stage was determined according to the most-recently published update.[14] The diagnosis of cirrhosis was based on histology or, if not available, established according to clinical, laboratory and radiology data, and the results of non-invasive tests (vibration-controlled transient elastography and/or the specialized blood test FibroMeter).

#### *Follow-up data*

The first HCC treatment performed after presentation at the multidisciplinary liver tumor meetings was collected. Therapies were categorized into four groups: i) curative (surgical resection, or percutaneous ablation); ii) endovascular (transarterial chemoembolization, or transarterial radioembolization); iii) systemic (tyrosine kinase inhibitors); or iv) best supportive care. Dates of death were obtained from the patient files or by consulting the national French Birth and Death Registry. Follow-up ended in May 2022. Overall survival (OS) was defined as the time from the day of presentation at the multidisciplinary liver tumor meetings to death from any cause or the date of the last follow-up if the patient was still alive.

### Statistics

Quantitative variables were expressed as medians with first and third quartiles and qualitative variables as percentages. Comparisons were performed using the Mann-Whitney test for quantitative variables and the Fisher's exact test for qualitative variables. OS curves were determined using the Kaplan-Meier method and compared with the Log-rank test. Variables with $p$ values <0.10 in univariate analysis (univariate Cox Model for quantitative variables, Log-rank test for qualitative variables) were introduced in a multivariate Cox model (forward stepwise selection) to identify the independent predictors of OS. Two multivariate analyses were performed: a first one including individual parameters, and a second one where the composite BCLC score was introduced without the individual parameters it includes. Statistical analyses were performed using SPSS version 25.0 software (IBM, Armonk, NY, USA).

## Results

### Patient and HCC characteristics

In all, 354 patients with MASLD-related HCC were included (Angers: n = 172, Bobigny: n = 91, Creteil: n = 46, and Paris Saint Antoine: n = 45). HCC occurred with underlying cirrhosis in 230 (65%) patients and without it in 124 (35%). HCC was diagnosed histologically in 61% of patients overall, and specifically in 49% of patients with cirrhosis *vs.* 81% of those without ($p$ <0.001). HCC was detected via screening in 60% of patients with cirrhosis *vs.* only 7% of patients without it ($p$ <0.001). Among patients without cirrhosis, diagnosis was mostly incidental (72%), as expected. Compared to patients with cirrhosis, those without it were significantly older, more frequently men, less frequently diabetic, and had better liver function tests (Table 1). Extra-tumor liver histology was available for 126 (55%) patients with cirrhosis and 104 (84%) without. Fibrosis stages were F0 in 13 non-cirrhotic patients, F1 in 17, F2 in 23, and F3 in 31 (data was missing for fibrosis stage in 20 patients). Patients without cirrhosis had large tumors (≥5 cm) more frequently than those with cirrhosis (55% *vs.* 28%, $p$ <0.001), and inversely, patients with cirrhosis more frequently presented with portal vein tumor thrombosis than those without it (25% *vs.* 14%, $p$ = 0.019). In both groups, half of the patients had localized HCC (BCLC stage 0 or A). However, the global distribution of BCLC stages significantly

Table 1. Patient and tumor characteristics.

| | All (N = 354) | Cirrhosis (n = 230) | No cirrhosis (n = 124) | *p* value |
|---|---|---|---|---|
| Age (years) | 73.0 (66.0–79.0) | 72.0 (65.8–79.0) | 75.0 (68.3–80.0) | 0.018 |
| Male sex (%) | 78.0 | 73.5 | 86.3 | 0.007 |
| BMI (kg/m$^2$) | 29.4 (26.1–33.0) | 29.6 (26.1–33.0) | 28.9 (26.3–32.4) | 0.379 |
| Diabetes (%) | 71.5 | 77.3 | 61.1 | 0.003 |
| Arterial hypertension (%) | 80.8 | 78.4 | 85.3 | 0.175 |
| Discovery (%) | | | | <0.001 |
| Incidental | 42.7 | 26.5 | 72.3 | |
| Clinical symptoms | 16.2 | 13.9 | 20.5 | |
| Screening | 41.0 | 59.6 | 7.2 | |
| Platelets (G/L) | 173 (116–247) | 137 (96–195) | 247 (189–310) | <0.001 |
| Bilirubin (µmol/L) | 13 (9–20) | 14 (10 –24) | 10.0 (7.0–16) | <0.001 |
| Prothrombin time (%) | 83 (72–94) | 78 (69–89) | 91 (80–99) | <0.001 |
| Creatinine (µmol/L) | 77 (65–94) | 74 (62–93) | 81 (69–96) | 0.039 |
| AFP (ng/ml) | 9 (4–176) | 9 (4–187) | 8 (3–167) | 0.178 |
| AFP (%): | | | | 0.334 |
| ≤100 ng/ml | 69.8 | 68.9 | 71.4 | |
| 101–1,000 ng/ml | 14.8 | 13.6 | 16.8 | |
| >1,000 ng/ml | 15.4 | 17.5 | 11.8 | |
| Liver involvement (%) | | | | 0.118 |
| One lobe | 87.5 | 85.1 | 92.1 | |
| Both lobes | 12.5 | 14.9 | 7.9 | |
| Number of lesions (%) | | | | 0.310 |
| 1 | 52.9 | 50.9 | 56.7 | |
| 2–3 | 24.9 | 27.5 | 20.0 | |
| ≥4 | 22.2 | 21.6 | 23.3 | |
| Size of the largest lesion (%) | | | | <0.001 |
| <3 cm | 34.0 | 42.9 | 17.0 | |
| 3–5 cm | 28.5 | 28.8 | 28.0 | |
| ≥5 cm | 37.5 | 28.3 | 55.0 | |
| Portal vein tumor thrombosis (%) | 20.7 | 24.6 | 13.7 | 0.019 |
| Extrahepatic metastasis (%) | 10.3 | 8.8 | 12.9 | 0.270 |
| BCLC classification (%) | | | | 0.028 |
| 0 | 8.9 | 11.1 | 4.9 | |
| A | 41.1 | 37.8 | 47.2 | |
| B | 19.3 | 17.8 | 22.0 | |
| C | 18.7 | 18.2 | 19.5 | |
| D | 12.1 | 15.1 | 6.5 | |
| Treatment (%) | | | | <0.001 |
| Surgery | 21.8 | 11.0 | 41.0 | |
| Percutaneous ablation | 27.1 | 35.8 | 11.5 | |
| TACE | 17.4 | 21.1 | 10.7 | |
| TARE | 7.1 | 5.5 | 9.8 | |
| Tyrosine kinase inhibitors | 7.9 | 5.5 | 12.3 | |
| Best supportive care | 18.8 | 21.1 | 14.8 | |

Statistical comparisons between the cirrhotic and non-cirrhotic patients were performed using the Mann-Whitney *U* test (for quantitative variables) or the Fisher's exact test (for qualitative variables).
AFP, alpha-foetoprotein; TACE, transarterial chemoembolization; TARE, transarterial radioembolization.

differed with notably more BCLC D in patients with cirrhosis (15% *vs.* 7%, $p = 0.024$), linked to impaired liver function.

## Factors predictive of overall survival

During follow-up, 182 patients with and 83 patients without cirrhosis died. Median OS was 29 months (95% CI 23–34), with 70% one-year survival and 45% three-year survival (Fig. S1). Patients with cirrhosis had worse OS than patients without it ($p = 0.043$, Fig. 1). The median OS was 28 months (95% CI 22–34) in patients with cirrhosis *vs.* 36 months (95% CI 22–51) for those without it. The other prognostic parameters in univariate analysis were age, bilirubin, prothrombin time, creatinine, alpha-foetoprotein, number of HCC lesions, size of the largest HCC lesion, presence of portal vein tumor thrombosis, presence of extrahepatic metastasis, and BCLC classification stage (Table S1).

Cirrhosis lost its independent association with OS in multivariate analysis (Table 2). In the multivariate analysis including only individual parameters, independent prognostic predictors were related to age, liver function (prothrombin time, bilirubin), and tumor burden (alpha-foetoprotein, number of lesions, size of the largest lesion, and portal vein tumor thrombosis). In the model including the BCLC classification without its composite parameters, independent predictors were age, alpha-foetoprotein and BCLC stage. OS remained similar between BCLC stages 0 (median: 55 months, 95% CI 26–84) and A (median: 53 months, 95% CI 37–69), then progressively

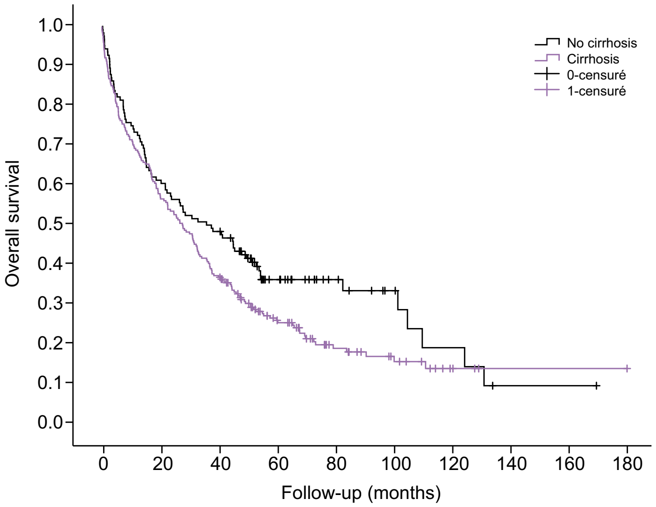


**Fig. 1. Overall survival according to cirrhosis status.** Statistical comparison between patients with and without cirrhosis was conducted using the log-rank test.

declined over stages B (median: 29 months, 95% CI 23–35), C (median: 12 months, 95% CI 6–19) and D (median: 2 months, 95% CI 1–3; Fig. 2A). Median OS in the three groups delineated by serum levels of alpha-foetoprotein (≤100, 101–1,000, >1,000 ng/ml) were respectively: 43 months (95% CI 36–49), 15 months (95% CI 11–20), and 5 months (95% CI 1–8) (Fig. 2B).

## Treatment of hepatocellular carcinoma

Of the study's patients, 49% received curative-intent treatment (resection: 22%, percutaneous ablation: 27%), 24% endovascular treatment (transarterial chemoembolization: 17%; transarterial radioembolization: 7%), 8% systemic treatment (all tyrosine kinase inhibitors), and 19% best supportive care. As expected, OS was strongly related to treatment modalities (Fig. S2). The rate of curative treatment was similar between patients with and without cirrhosis (respectively: 47% *vs.* 52%, $p$ = 0.366). However, treatment modalities differed between these two groups, with more surgery (41% *vs.* 11%, $p$ <0.001), less percutaneous ablation (11% *vs.* 36%, $p$ <0.001), less transarterial chemoembolization (11% *vs.* 21%, $p$ = 0.017) and

**Table 2. Independent predictors of overall survival.**

| | Univariate | Multivariate | |
|---|---|---|---|
| **Variables** | ***p* value** | ***p* value** | **aHR (95% CI)** |
| **Individual parameters** | | | |
| Age (years) | <0.001 | <0.001 | 1.04 [1.02–1.07] |
| Underlying cirrhosis (%) | 0.043 | 0.200 | – |
| Bilirubin (µmol/L) | <0.001 | <0.001 | 1.01 [1.01–1.02] |
| Prothrombin time (%) | 0.016 | 0.002 | 0.99 [0.98–1.00] |
| Creatinine (µmol/L) | 0.035 | 0.340 | – |
| Alpha-foetoprotein (ng/ml) | <0.001 | 0.037 | |
| ≤100 | | | 1.0 (ref) |
| 101–1,000 | | 0.725 | 1.09 [0.68–1.74] |
| >1,000 | | 0.010 | 1.87 [1.16–3.03] |
| Number of lesions (%) | <0.001 | 0.005 | |
| 1 | | | 1.0 (ref) |
| 2–3 | | 0.005 | 1.64 [1.16–2.33] |
| ≥4 | | 0.018 | 1.87 [1.11–3.15] |
| Size of the largest lesion (%) | <0.001 | <0.001 | |
| <3 cm | | | 1.0 (ref) |
| 3–5 cm | | 0.443 | 1.17 [0.78–1.76] |
| ≥5 cm | | <0.001 | 2.06 [1.41–3.01] |
| Portal vein tumor thrombosis (%) | <0.001 | 0.004 | 1.98 [1.25–3.15] |
| Extrahepatic metastasis (%) | <0.001 | 0.705 | – |
| **With BCLC classification** | | | |
| Age (years) | <0.001 | <0.001 | 1.04 [1.03–1.06] |
| Underlying cirrhosis (%) | 0.043 | 0.371 | – |
| Creatinine (µmol/L) | 0.035 | 0.211 | – |
| Alpha-foetoprotein (ng/ml) | <0.001 | 0.003 | |
| ≤100 | | – | 1.0 (ref) |
| 101–1,000 | | 0.228 | 1.26 [0.87–1.82] |
| >1,000 | | 0.001 | 1.94 [1.33–2.83] |
| BCLC stages | <0.001 | <0.001 | |
| 0 | | – | 1.0 (ref) |
| A | | 0.602 | 0.86 [0.49–1.51] |
| B | | 0.072 | 1.72 [0.95–3.09] |
| C | | 0.001 | 2.81 [1.55–5.09] |
| D | | <0.001 | 10.24 [5.26–19.90] |

Two different multivariate Cox Models were performed. The first model included the individual parameters that were significant in univariate analysis ($p$ <0.10). In this model, the BCLC classification which includes several of those parameters was not introduced. The second model included the BCLC classification while removing the individual parameters already used to define the BCLC stages (bilirubin, prothrombin time, number of lesions, size of the largest lesion, portal vein tumor thrombosis, extrahepatic metastasis). Variables with $p$ <0.10 in univariate analysis (univariate Cox Model for quantitative variables, Log-rank test for qualitative variables) were entered into multivariate Cox model (forward stepwise selection) with OR and 95% CIs calculated. A two-tailed $p$ value of <0.05 was considered statistically significant.
BCLC, Barcelona Clinic Liver Cancer.

more systemic treatment (12% *vs.* 6%, $p$ = 0.035) in patients without cirrhosis (Fig. 3A).

As expected, treatment modalities were strongly associated with BCLC classification stages (Fig. S3). Fig. 3B shows treatments according to BCLC stages and cirrhosis status. Percutaneous ablation was the treatment of choice in BCLC 0 stage, whereas surgery was mostly performed in patients with BCLC A without underlying cirrhosis. In the setting of large or numerous lesions, surgery was also more often performed in patients without cirrhosis than in those with it (Fig. 4). In cases where the largest lesion was ≥5 cm, surgery was performed in 42% of the patients without cirrhosis *vs.* 14% of the patients with cirrhosis ($p$ = 0.002, Fig. 4A). In cases of multiple lesions (≥4), surgery was performed in 19% of the patients without *vs.* 2% of those with cirrhosis ($p$ = 0.024, Fig. 4B). Importantly, OS was not significantly different between patients with and without cirrhosis who underwent surgery ($p$ = 0.074, Fig. S4).

### Trends in MASLD-related HCC over time

The number of cases of MASLD-related HCC increased and the ratio between patients with and without cirrhosis remained stable over the study period (Fig. 5). Etiologies were available for all HCC presented at the Angers multidisciplinary liver tumor board during the study period, enabling the study of trends in the prevalence of MASLD-related HCC over time. Between 2007 and 2018, 1,304 new patients with HCC were presented at the meeting (Fig. S5A). Excessive alcohol consumption was the main cause of chronic liver disease related to HCC (67.5%), followed by MASLD (13.4%), chronic hepatitis C (8.7%) and chronic hepatitis B (2.6%). Trends over the years showed that the rate of alcohol-related HCC remained stable (Fig. S5B). In contrast, MASLD-related HCC showed the most dramatic rise, from 5.6% of cases in 2007 to 19.0% in 2018, thereby becoming the second-leading cause of HCC.

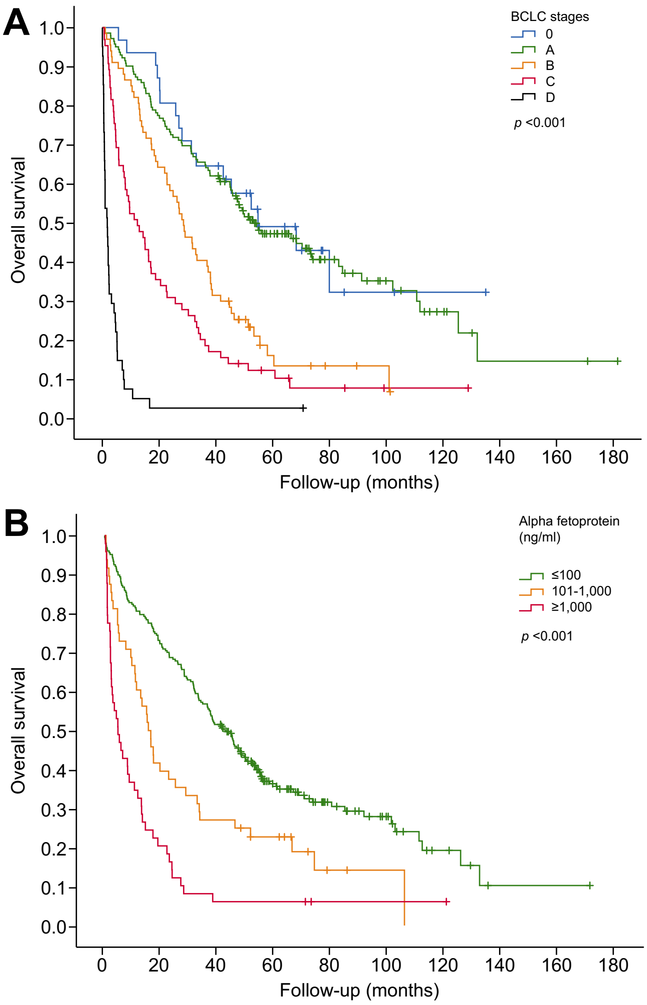


**Fig. 2. Overall survival as a function of BCLC stages (A) and alpha-foetoprotein serum level (B).** Statistical comparison between the groups was conducted using the log-rank test. BCLC, Barcelona Clinic Liver Cancer.

## Discussion

MASLD has become the leading cause of chronic liver disease worldwide and, consequently, the prevalence of MASLD-related HCC has increased over the last decades.[15,16] The results we present here confirm that MASLD-related HCC outside of cirrhosis is frequent (35% of cases) and that it tends to be diagnosed belatedly and with larger tumors. However, due to better liver function allowing for more aggressive therapy, patients with non-cirrhotic MASLD-related HCC had better prognoses than their counterparts with cirrhosis. Our study has several strengths including: i) a multicenter design with a large sample size of MASLD-related HCC unequivocally diagnosed by dedicated multidisciplinary liver tumor boards; ii) a long study period; and iii) the availability of the French Birth and

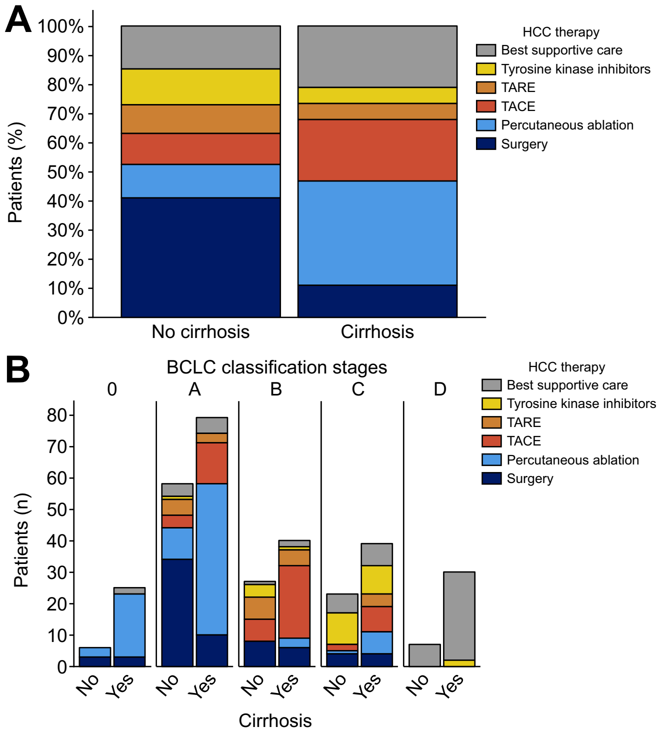


**Fig. 3. Treatment modalities for HCC as a function of cirrhosis status (A), and as a function of both cirrhosis status and BCLC stages (B).** Statistical comparisons between the groups were performed using the Mann-Whitney *U* test (for quantitative variables) or the Fisher's exact test (for qualitative variables). BCLC, Barcelona Clinic Liver Cancer; HCC, hepatocellular carcinoma; TACE, transarterial chemoembolization; TARE, transarterial radioembolization.

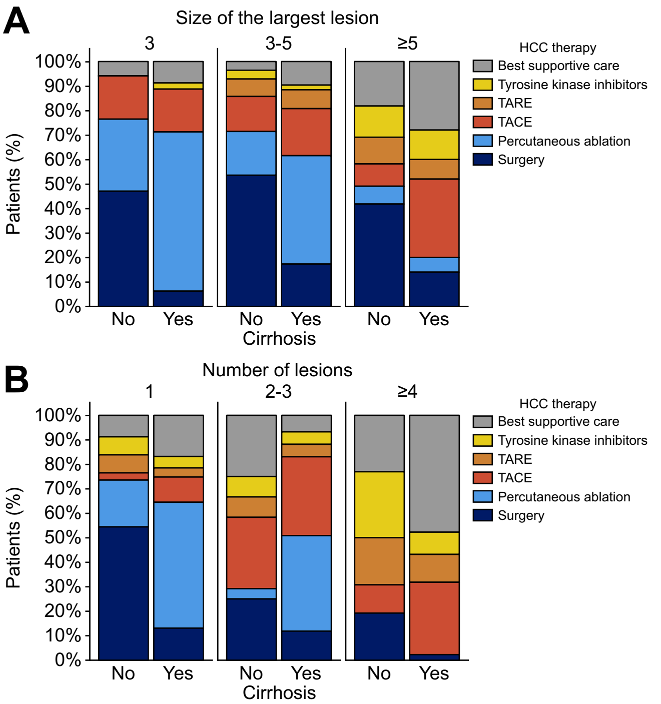


**Fig. 4. Treatment modalities for HCC as a function of cirrhosis status and the size of the largest lesion or the number of lesions.** Statistical comparisons between the groups were performed using the Fisher's exact test. (A) In cases where the largest lesion was ≥5 cm, surgery was performed in 42% of the patients without cirrhosis *vs.* 14% of those with cirrhosis ($p$ = 0.002). In cases with largest lesion between 3–5 cm and those with largest lesion <3 cm, surgery was performed respectively in 54% *vs.* 17% ($p$ = 0.002) and 47% *vs.* 6% ($p$ <0.001). (B) In case of ≥4 lesions, surgery was performed in 19% of the patients without cirrhosis *vs.* 2% of those with cirrhosis ($p$ = 0.024). Among patients with 2–3 lesions and those with one lesion, surgery was performed respectively in: 25% *vs.* 12% ($p$ = 0.183) and 54% *vs.* 13% ($p$ <0.001). BCLC, Barcelona Clinic Liver Cancer; HCC, hepatocellular carcinoma; TACE, transarterial chemoembolization; TARE, transarterial radioembolization.

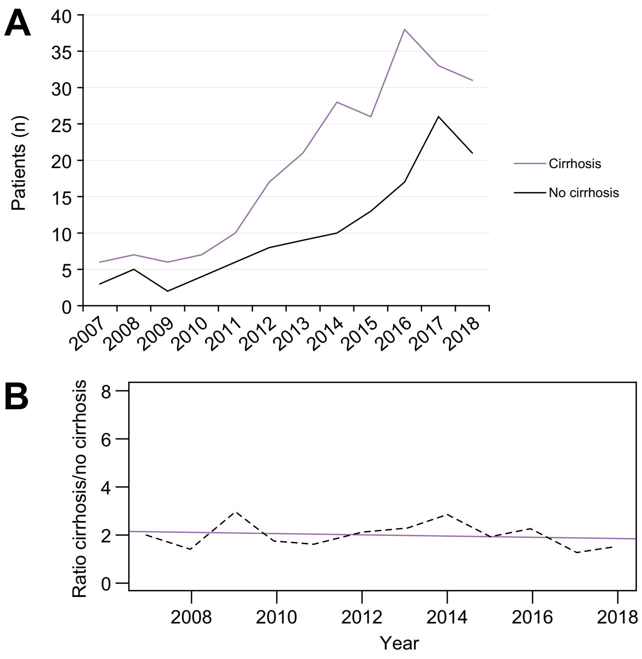


**Fig. 5. Trends in the number of MASLD-related hepatocellular carcinomas presented at the multidisciplinary liver tumor meetings (A) and in the cirrhosis/no cirrhosis ratio over the study period (B).** In (B) the number of MASLD-related hepatocellular carcinomas is indicated with the dashed curve, and the blue line represents the regression line of the ratio cirrhosis/no cirrhosis. MASLD, metabolic dysfunction-associated steatotic liver disease.

Death Registry, which ensured no loss of follow-up concerning deaths.

Previous studies have reported that only one-third of patients with MASLD-related HCC were in a screening program for HCC before cancer diagnosis, *vs.* 56% of patients with HCC secondary to other causes of chronic liver disease.[9] This rate was recently evaluated at 45% in 1,181 patients with MASLD-related HCC in the Italian ITA.LI.CA national registry.[17] We found a similar proportion in our study (41%), but also, and as expected, that the diagnosis of HCC via screening dropped dramatically to only 7% in patients without cirrhosis. Indeed, biannual screening for HCC is recommended for patients with cirrhosis[18,19] but not for those without it. It is thus not surprising that the rate of incidental diagnoses of HCC in patients without cirrhosis was so high (72%). Consequently, patients with HCC occurring without cirrhosis were older and had larger tumors than those with cirrhosis. The pathophysiology driving HCC occurrence in non-cirrhotic MASLD livers remains largely unknown. It has recently been suggested that the MASLD phenotype, rather than obesity itself, is associated with cancer and in particular HCC.[20] Many mechanisms may be involved in the occurrence of HCC in non-cirrhotic MASLD, including insulin-resistance, low-grade chronic systemic inflammation, adipokine dysregulation, local metabolic stress, gut microbiota dysbiosis, immune dysregulation, and sex hormones.[21] There is an urgent need to deepen our understanding of the pathogenesis of HCC in non-cirrhotic MASLD, to better define the profile of patients at risk and to discover new biomarkers, with the aim of improving the early diagnosis of HCC occurring outside cirrhosis in patients with MASLD.

In univariate analysis, patients with cirrhosis had worse prognoses than those without in our multicenter cohort. However, the association between cirrhosis and OS lost its significance in multivariate analysis where the independent prognostic predictors were related to liver function, tumor burden and age. On one hand, our patients without cirrhosis were older and had larger tumors, both of which are associated with worse survival. On the other, they showed preserved liver function, which undoubtedly contributed to their better survival compared to patients with cirrhosis. Those poor prognostic factors in patients without cirrhosis may thus have been counterbalanced by the preserved liver function, which maintained surgical eligibility for some large or numerous tumor cases. In a recent meta-analysis, the rate of MASLD-related HCC assigned to curative therapy was evaluated at 65%, with 34% of patients undergoing surgery.[9] In the 2022 updated analysis of the ITA.LI.CA registry, curative therapy was reported in 47% of cases, and surgery in 19%.[17] We also found a high 50% rate of curative-intent treatment in our cohort, with no significant difference between patients with and without cirrhosis. Importantly though, the pattern of curative treatment was different between these two groups, with percutaneous ablation performed more frequently in patients with cirrhosis (36% *vs.* 11%), and surgery performed more frequently in patients without cirrhosis (41% *vs.* 11%). A monocentric study

performed in Sweden on 225 patients with MASLD-related HCC (142 with cirrhosis, 83 without) reported comparable results.[22] In that work, 35% and 5% of patients without cirrhosis underwent surgery and percutaneous ablation, respectively, *vs.* 8% and 20% of patients with cirrhosis. However, characteristics of the tumors (size and number) as a function of cirrhosis status in patients undergoing surgery were not detailed in the Swedish study. In our cohort, patients without cirrhosis were more likely to undergo surgery even in cases of large tumors or numerous ≥4 tumors. Such aggressive management seemed justified as OS in the surgery group was not significantly different between patients with and without cirrhosis.

The annual proportion of MASLD-related HCC has been shown to be stable in some studies. For example, an American study on 1,500 patients with HCC in Veterans Administration Hospitals reported yearly proportions between 7.5% and 12.0% in the 2005–2010 period.[10] In contrast, more recent studies have highlighted a significant increase in the rate of MASLD-related HCC over the last two decades in the UK,[23] Sweden,[22] and Italy,[17] with MASLD becoming the second or even the primary cause of chronic liver disease underlying HCC. Using the methodology framework of the Global Burden of Disease study, a recent work showed that MASLD was the fastest growing etiology of incident liver cancer (+39%) and liver cancer deaths (+38%) worldwide between 2010 and 2019.[16] The age-standardized incident rate for MASLD-related HCC increased in five of the six WHO regions, with the greatest increase in the Americas. The results from our multicenter French cohort align with these data. We found not only a significant increase in MASLD-related HCC but also that MASLD had become the second most-frequent liver disease underlying HCC in the Angers center, now accounting for one-fifth of the cases therein. Our analysis on underlying cirrhosis in MASLD-related HCC also showed that the cirrhosis/no cirrhosis ratio remained stable over the 12-year study period. A modification of this ratio, with an increase in the occurrence of MASLD-related HCC in the absence of cirrhosis, would have potentially suggested a recent superimposing action of a new "extra-metabolic co-factor", for example a new toxin or pollutant in the patient exposome. However, that is not what we observed in our work, and the stable ratio suggests that the observed increase in MASLD-related HCC without cirrhosis parallels, and is only a consequence of, the increasing burden of obesity and MASLD.

Our study has some limitations. The diagnosis of MASLD was only declarative and directly collected from the reports within the databases of the multidisciplinary liver tumor meetings. Nonetheless, those electronic reports were completed by specialists in hepatology from tertiary liver centers, an aspect that ensures accurate diagnoses. Some parameters were not available in a sufficient number of patients. For example, this was the case for albumin, which prevented us from including the ALBI score in our work. Finally, the etiology of all cases of HCC presented at the multidisciplinary meetings during the study period was only available at the Angers center, allowing only a monocentric assessment of the trend in causes of chronic liver disease underlying HCC. The survival we observed in patients with BCLC 0 and A was poorer than that previously reported in the literature. It should be remembered that our study population was old (median age 73 years) and, due to the MASLD context, enriched with a high burden of metabolic comorbidities with more than two-thirds of patients having type 2 diabetes and 80% arterial hypertension. All these factors will have contributed to the poor outcomes observed in BCLC 0 and A stages despite most of these patients being offered curative treatment.

In conclusion, the growing incidence of MASLD-related HCC brings new challenges for clinical practice because in 35% of cases, it occurs in the absence of underlying cirrhosis. Patients with MASLD-related HCC without cirrhosis are older and have larger tumors. However, they also have better liver function, which enables more aggressive management and greater recourse to surgery in advanced tumors. A better understanding of the pathophysiological processes driving HCC occurrence in non-cirrhotic MASLD is mandatory, a benefit of such increased knowledge would be the identification of new biomarkers for the screening and early management of these patients.


### Affiliations

[1]*Service d'Hépato-Gastroentérologie et Oncologie Digestive, Centre Hospitalier Universitaire d'Angers, Angers, France;* [2]*Laboratoire HIFIH UPRES EA3859, SFR 4208, Université d'Angers, Angers, France;* [3]*Service d'Hépatologie et Oncologie hépatique, AP-HP Sorbonne Paris Nord, Hôpital Universitaire Avicenne, Bobigny, France;* [4]*Service d'Hépatologie, AP-HP Saint-Antoine, France;* [5]*Service d'Hépatologie, AP-HP Henri Mondor, Créteil, France;* [6]*Service d'Anatomie et de Cytologie Pathologiques, APHP Sorbonne Paris Nord, Hôpital Universitaire Avicenne, Bobigny, France;* [7]*Centre de recherche des Cordeliers, Sorbonne Université, Inserm UMR-1162, Université de Paris, team «Functional Genomics of Solid Tumors», Equipe labellisée Ligue Nationale Contre le Cancer, Labex OncoImmunology, F-75006 Paris, France*


### Abbreviations

BCLC, Barcelona Clinic Liver Cancer; HC, hepatocellular carcinoma; MASLD, metabolic dysfunction-associated steatotic liver disease; NAFLD, non-alcoholic fatty liver disease; OS, overall survival.


### Financial support

The authors did not receive any financial support to produce this manuscript.


### Conflict of interest

The authors of this study declare that they do not have any conflict of interest.

Please refer to the accompanying ICMJE disclosure forms for further details.

### Authors' contributions

Study design: Carole Vitellius, Elvire Desjonqueres, Jerome Boursier, Nathalie Ganne-Carrie. Data acquisition: all authors. Analysis: Carole Vitellius, Elvire Desjonqueres, Jerome Boursier, Nathalie Ganne-Carrie. Drafting/critical revision: Carole Vitellius, Elvire Desjonqueres, Marie Lequoy, Frederic Oberti, Jerome Boursier, Nathalie Ganne-Carrie.

### Data availability statement

The data that support the findings of this study are available from the corresponding author upon request.

### Supplementary data

Supplementary data to this article can be found online at https://doi.org/10.1016/j.jhepr.2024.101160.

### References


[1] Sung H, Ferlay J, Siegel RL, et al. Global cancer statistics 2020: GLOBOCAN estimates of incidence and mortality worldwide for 36 cancers in 185 countries. CA Cancer J Clin 2021;71:209–249.

[2] Huang DQ, El-Serag HB, Loomba R. Global epidemiology of NAFLD-related HCC: trends, predictions, risk factors and prevention. Nat Rev Gastroenterol Hepatol 2021;18:223–238.
[3] Rinella ME, Lazarus JV, Ratziu V, et al. A multisociety Delphi consensus statement on new fatty liver disease nomenclature. J Hepatol 2023;79:1542–1556.
[4] Younossi ZM, Golabi P, Paik JM, et al. The global epidemiology of nonalcoholic fatty liver disease (NAFLD) and nonalcoholic steatohepatitis (NASH): a systematic review. Hepatology 2023;77:1335–1347.
[5] Younossi ZM, Koenig AB, Abdelatif D, et al. Global epidemiology of nonalcoholic fatty liver disease-Meta-analytic assessment of prevalence, incidence, and outcomes. Hepatology 2016;64:73–84.
[6] Diehl AM, Day C. Nonalcoholic steatohepatitis. N Engl J Med 2018;378:781.
[7] Estes C, Anstee QM, Arias-Loste MT, et al. Modeling NAFLD disease burden in China, France, Germany, Italy, Japan, Spain, United Kingdom, and United States for the period 2016-2030. J Hepatol 2018;69:896–904.
[8] Wong CR, Njei B, Nguyen MH, et al. Survival after treatment with curative intent for hepatocellular carcinoma among patients with vs without non-alcoholic fatty liver disease. Aliment Pharmacol Ther 2017;46:1061–1069.
[9] Tan DJH, Ng CH, Lin SY, et al. Clinical characteristics, surveillance, treatment allocation, and outcomes of non-alcoholic fatty liver disease-related hepatocellular carcinoma: a systematic review and meta-analysis. Lancet Oncol 2022;23:521–530.
[10] Mittal S, Sada YH, El-Serag HB, et al. Temporal trends of nonalcoholic fatty liver disease-related hepatocellular carcinoma in the veteran affairs population. Clin Gastroenterol Hepatol 2015;13:594–601 e591.
[11] Stine JG, Wentworth BJ, Zimmet A, et al. Systematic review with meta-analysis: risk of hepatocellular carcinoma in non-alcoholic steatohepatitis without cirrhosis compared to other liver diseases. Aliment Pharmacol Ther 2018;48:696–703.
[12] Canivet CM, Boursier J, Loomba R. New nomenclature for nonalcoholic fatty liver disease: understanding metabolic dysfunction-associated steatotic liver disease, metabolic dysfunction- and alcohol-associated liver disease, and their implications in clinical practice. Semin Liver Dis 2024;44:35–42.
[13] Kleiner DE, Brunt EM, Van Natta M, et al. Design and validation of a histological scoring system for nonalcoholic fatty liver disease. Hepatology 2005;41:1313–1321.
[14] Reig M, Forner A, Rimola J, et al. BCLC strategy for prognosis prediction and treatment recommendation: the 2022 update. J Hepatol 2022;76:681–693.
[15] Wong VW, Ekstedt M, Wong GL, Hagstrom H. Changing epidemiology, global trends and implications for outcomes of NAFLD. J Hepatol 2023;79:842–852.
[16] Huang DQ, Singal AG, Kono Y, et al. Changing global epidemiology of liver cancer from 2010 to 2019: NASH is the fastest growing cause of liver cancer. Cell Metab 2022;34:969–977 e962.
[17] Vitale A, Svegliati-Baroni G, Ortolani A, et al. Epidemiological trends and trajectories of MAFLD-associated hepatocellular carcinoma 2002-2033: the ITA.LI.CA database. Gut 2023;72:141–152.
[18] Singal AG, Llovet JM, Yarchoan M, et al. AASLD practice guidance on prevention, diagnosis, and treatment of hepatocellular carcinoma. Hepatology 2023;78:1922–1965.
[19] Liver. EAftSot. EASL clinical practice guidelines: management of hepatocellular carcinoma. J Hepatol 2018;69:182–236.
[20] Allen AM, Hicks SB, Mara KC, et al. The risk of incident extrahepatic cancers is higher in non-alcoholic fatty liver disease than obesity - a longitudinal cohort study. J Hepatol 2019;71:1229–1236.
[21] Polyzos SA, Chrysavgis L, Vachliotis ID, et al. Nonalcoholic fatty liver disease and hepatocellular carcinoma:Insights in epidemiology, pathogenesis, imaging, prevention and therapy. Semin Cancer Biol 2023;93:20–35.
[22] Bengtsson B, Stal P, Wahlin S, et al. Characteristics and outcome of hepatocellular carcinoma in patients with NAFLD without cirrhosis. Liver Int 2019;39:1098–1108.
[23] Dyson J, Jaques B, Chattopadyhay D, et al. Hepatocellular cancer: the impact of obesity, type 2 diabetes and a multidisciplinary team. J Hepatol 2014;60:110–117.

Keywords: hepatocarcinoma; steatotic liver disease; NAFLD; survival.
*Received 31 January 2024; received in revised form 19 June 2024; accepted 25 June 2024; Available online 29 June 2024*

Journal of Hepatology, Volume *6*

## Supplemental information

# MASLD-related HCC: Multicenter study comparing patients with and without cirrhosis

**Carole Vitellius, Elvire Desjonqueres, Marie Lequoy, Giuliana Amaddeo, Isabelle Fouchard, Gisele N'Kontchou, Clemence M. Canivet, Marianne Ziol, Hélène Regnault, Adrien Lannes, Frederic Oberti, Jerome Boursier, and Nathalie Ganne-Carrie**

# MASLD-related HCC: Multicenter study comparing patients with and without cirrhosis

Carole Vitellius, Elvire Desjonqueres, Marie Lequoy, Giuliana Amaddeo, Isabelle Fouchard, Gisele N'Kontchou, Clemence M Canivet, Marianne Ziol, Hélène Regnault, Adrien Lannes, Frederic Oberti, Jerome Boursier, Nathalie Ganne-Carrie

Table of contents

Table S1 .......... 2

Fig. S1 .......... 3

Fig. S2 .......... 4

Fig. S3 .......... 5

Fig. S4 .......... 6

Fig. S5. .......... 7

**Table S1:** Predictors of overall survival in univariate analysis

| | All (n=354) | Alive (n=89) | Dead (n=265) | p |
|---|---|---|---|---|
| Centre (%): | | | | 0.435 |
| Angers | 48.6 | 42.7 | 50.6 | |
| Bondy | 25.7 | 27.0 | 25.3 | |
| Creteil | 13.0 | 13.5 | 12.8 | |
| Paris Saint Antoine | 12.7 | 16.9 | 11.3 | |
| Age (years) | 73.0 (66.0–79.0) | 71.0 (62.0–77.0) | 73.0 (68.0–80.0) | <0.001 |
| Male sex (%) | 78.0 | 77.5 | 78.1 | 0.715 |
| BMI (kg/m$^2$) | 29.4 (26.1–33.0) | 28.9 (26.1–32.7) | 29.6 (26.1–33.1) | 0.441 |
| Diabetes (%) | 71.5 | 69.6 | 72.2 | 0.362 |
| Arterial hypertension (%) | 80.8 | 80.0 | 81.1 | 0.980 |
| Underlying cirrhosis (%) | 65.0 | 53.9 | 68.7 | 0.043 |
| Platelets (G/l) | 173 (116–247) | 186 (118–248) | 172 (116–247) | 0.177 |
| Bilirubin (µmol/l) | 13 (9–20) | 10 (7–15) | 13 (9–22) | <0.001 |
| Prothrombin time (%) | 83 (72–94) | 85 (75–96) | 82 (72–93) | 0.016 |
| Creatinine (µmol/l) | 77 (65–94) | 76 (65–88) | 78 (65–96) | 0.035 |
| AFP (ng/ml) | 9 (4–176) | 5 (3–14) | 16 (5–381) | 0.002 |
| AFP (%): | | | | <0.001 |
| ≤100 ng/ml | 69.8 | 86.7 | 64.0 | |
| 101–1000 ng/ml | 14.8 | 9.6 | 16.5 | |
| >1000 ng/ml | 15.4 | 3.6 | 19.4 | |
| Number of lesions (%): | | | | <0.001 |
| 1 | 52.9 | 67.8 | 47.8 | |
| 2–3 | 24.9 | 25.3 | 24.7 | |
| ≥4 | 22.2 | 6.9 | 27.5 | |
| Size of the largest lesion (%): | | | | <0.001 |
| <3 cm | 34.0 | 47.7 | 28.3 | |
| 3–5 cm | 28.5 | 30.2 | 27.8 | |
| ≥5 cm | 37.5 | 22.1 | 43.9 | |
| Portal vein tumor thrombosis (%) | 20.7 | 6.7 | 25.5 | <0.001 |
| Extra-hepatic metastasis (%) | 10.3 | 3.4 | 12.6 | <0.001 |
| BCLC classification (%): | | | | <0.001 |
| 0 | 8.9 | 15.9 | 6.5 | |
| A | 41.1 | 63.6 | 33.5 | |
| B | 19.3 | 12.5 | 21.5 | |
| C | 18.7 | 6.8 | 22.7 | |
| D | 12.1 | 1.1 | 15.8 | |

BMI: body mass index, AFP: alpha-foetoprotein

Statistical comparisons between the groups were performed using the univariate Cox Model for quantitative variables and log-rank test for qualitative variables.

**Fig. S1:** Overall survival in the study population

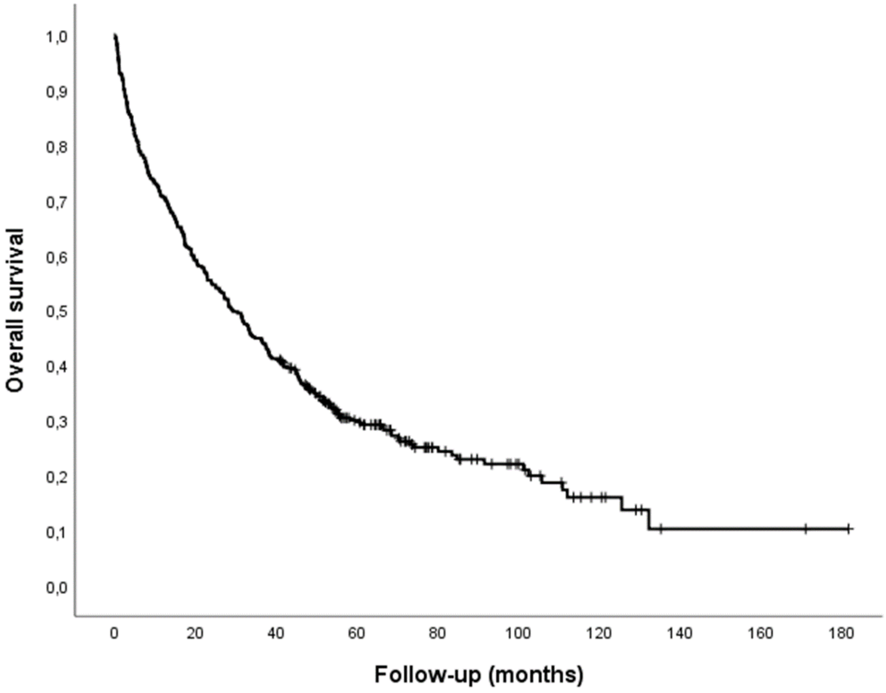

**Fig. S2:** Overall survival according to treatment modalities for hepatocellular carcinoma

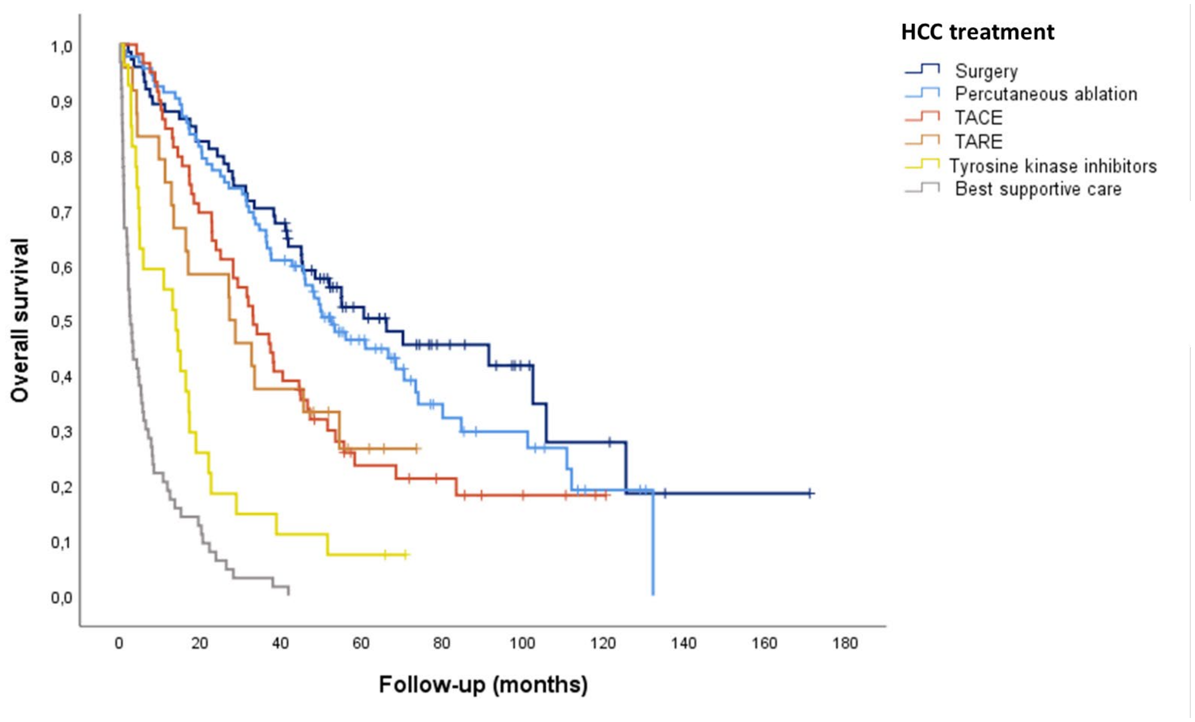

**Fig. S3:** Treatment modalities for hepatocellular carcinoma according to BCLC classification stages

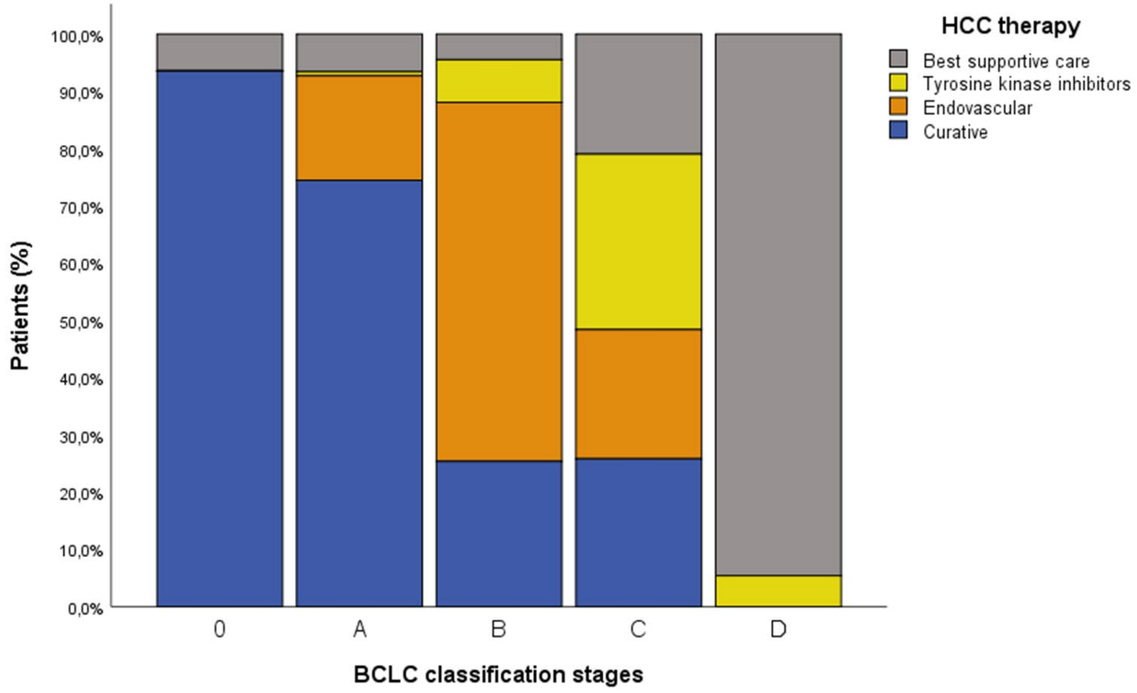

**Fig. S4:** Overall survival in patients undergoing surgery according to cirrhosis status

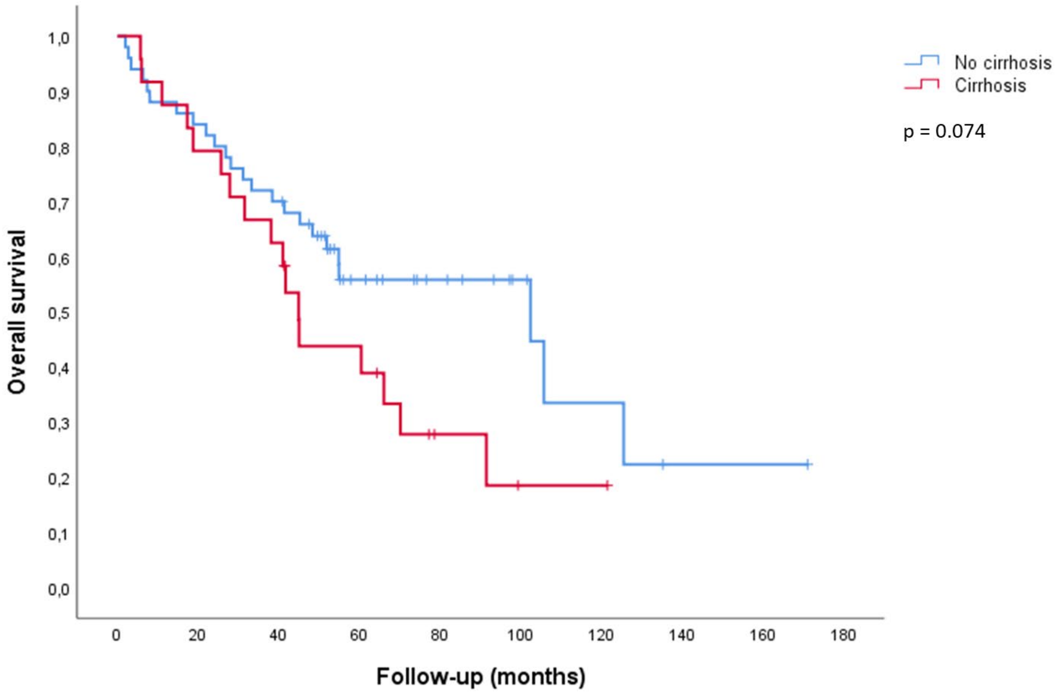


Statistical comparison between the cirrhotic and non-cirrhotic patients was conducted using the log-rank test.

**Fig. S5:** Number of hepatocellular carcinomas newly presented at the multidisciplinary liver tumor meeting of the Angers University Hospital between 2007 and 2018 (panel s5a), and trends in the causes of chronic liver disease associated with hepatocellular carcinoma in the Angers center (panel s5b).

**a**

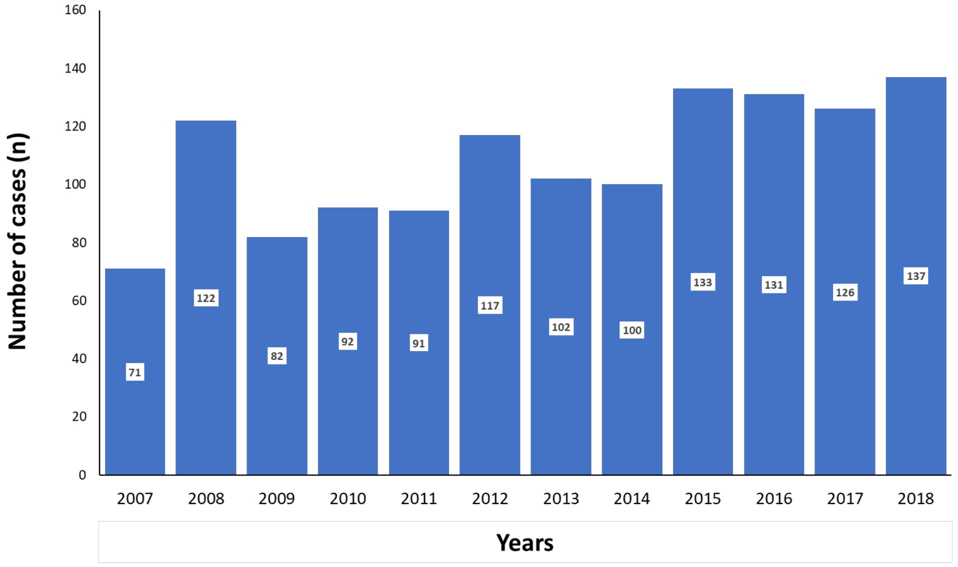


**b**

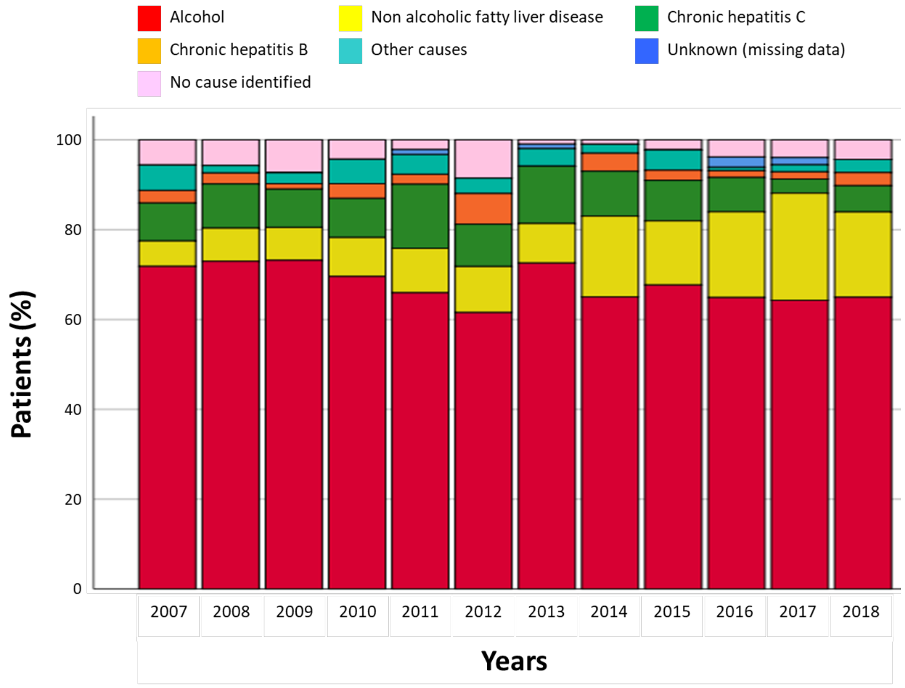

Supplement: Multimedia component 4 [file mmc4.pdf]
